# Application of MFI-5 in severe complications and unfavorable outcomes after radical resection of colorectal cancer

**DOI:** 10.1186/s12957-023-03186-4

**Published:** 2023-09-26

**Authors:** Lihong Huang, Zhifa Li, Mengru Jian, Xiaobing Wu, Huixian Chen, Haifeng Qin, Ziqiao Li, Shixi Song, Yingjun Xie, Rong Chen

**Affiliations:** 1 Gastrointestinal Surgery; Guangdong Provincial Key Laboratory of Major Obstetric Diseases; Guangdong Provincial Clinical Research Center for Obstetrics and Gynecology, The Third Affiliated Hospital of Guangzhou Medical University, Guangzhou, 510150 China; 2 Department of Clinical Medicine, The Third Clinical School of Guangzhou Medical University, Guangdong Province, 510150 China; 3 Department of Obstetrics and Gynecology; Guangdong Provincial Key Laboratory of Major Obstetric Diseases; Guangdong Provincial Clinical Research Center for Obstetrics and Gynecology; Guangdong-Hong Kong-Macao Greater Bay Area Higher Education Joint Laboratory of Maternal-Fetal Medicine, The Third Affiliated Hospital of Guangzhou Medical University, Guangzhou, 510150 China

**Keywords:** Radical resection of colorectal carcinoma, mFI-5, Postoperative Complications, Frailty, Risk stratification

## Abstract

**Background:**

Frailty is considered a characteristic manifestation of physiological decline in multiple organ systems, which significantly increases the vulnerability of elderly individuals with colorectal cancer (CRC) and is associated with a poor prognosis. While studies have demonstrated that the 11-factor Modified Frailty Index (mFI-11) can effectively predict adverse outcomes following radical resection of CRC, there is a lack of research on the applicability of the 5-factor Modified Frailty Index (mFI-5) within this patient population.

**Methods:**

In this retrospective analysis, we examined a cohort of CRC patients aged 65 years and above who had undergone radical resection. For each patient, we calculated their mFI-5 score, considering a score of ≥ 2 as an indication of frailty. We conducted univariate and multivariate analyses to assess the association between the mFI-5 and adverse outcomes as well as postoperative complications.

**Results:**

Patients with an mFI-5 score ≥ 2 exhibited a significantly higher incidence of serious postoperative complications (53% vs. 30%; *P* = 0.001) and experienced a longer hospital stay [19.00 (15.00–24.50) vs. 17.00 (14.00–20.00); *P* < 0.05]. Notably, an mFI-5 score greater than 2 emerged as an independent risk factor for severe postoperative complications (odds ratio: 2.297; 95% confidence interval: 1.216 to 4.339; *P* = 0.01). Furthermore, the mFI-5 score displayed predictive capabilities for severe postoperative complications with an area under the receiver operating characteristic (ROC) curve of 0.629 (95% confidence interval: 0.551 to 0.707; *P* < 0.05).

**Conclusion:**

The mFI-5 demonstrates a high level of sensitivity in predicting serious complications, prolonged hospital stays, and mortality following radical resection of colorectal carcinoma. As a practical clinical assessment tool, the mFI-5 enables the identification of high-risk patients and facilitates preoperative optimization.

**Supplementary Information:**

The online version contains supplementary material available at 10.1186/s12957-023-03186-4.

## Introduction

Colorectal cancer (CRC) is a digestive tract disease that poses a significant threat to human health. According to the latest statistics from 2020, it ranks third in terms of incidence rate and second in terms of mortality rate among malignancies worldwide [1]. Elderly patients often experience varying degrees of organ degeneration, and they are particularly susceptible to underlying diseases such as lung infections, coronary heart disease, reduced body reserves, surgical trauma, postoperative complications, and even death. These factors can have adverse effects on prognosis and recovery [2, 3]. Recent research by Tsutomu et al. has demonstrated a significant increase in postoperative complications and readmission rates among patients with sarcopenia, which is one of the manifestations of weakness [4]. Consequently, there is a need for preoperative risk stratification and perioperative clinical consultation specifically tailored to elderly CRC patients (age ≥ 65 years) to improve the prognosis of radical CRC surgery [5, 6].

To better capture the cumulative physical deterioration and deficits, the Canadian Study of Health and Aging introduced a less complex frailty index known as the Canadian Study of Health and Aging-Frailty Index (CSHA-FI) [6]. In 2013, a modified frailty index (mFI) was developed by aligning the CSHA-FI with 11 variables collected by the American College of Surgeons National Surgical Quality Improvement Program (ACS-NSQIP) [7]. Similar to the CSHA-FI, mFI-11 has been validated as a reliable predictor of postoperative outcomes [8–11]. More recently, a simpler and less time-consuming mFI-5 has been developed [12]. The mFI-5 has undergone validation and demonstrated significant agreement with the mFI-11 in the context of upper gastrointestinal and multiorgan resections [13, 14]. The five comorbidities included in the mFI-5 are diabetes, congestive heart failure (CHF), chronic obstructive pulmonary disease (COPD) or pneumonia, hypertension requiring medication, and non-independent functional status. Each preoperative comorbidity is assigned a value of 1, resulting in total values ranging from 0 to 5. In terms of predicting poor postoperative prognosis, the mFI-5 has been validated and shown to have significant agreement with the mFI-11 in the context of upper orthopedics and multiorgan resections [15–19]. Early identification of frail patients and prompt intervention for diagnosis and treatment align with the theory of perioperative applications aimed at enhancing postoperative recovery [20, 21].

In numerous surgical areas, preoperatively assessable metrics have been found to significantly correlate with important postoperative metrics, such as hospital length of stay (LOS), cost, and unplanned readmissions [2, 22–24]. While previous assessment models have provided guidance, their limited clinical integration and slow implementation in practice have severely restricted their practical utility. In contrast, mFI-5, which can be readily identified in clinical screening settings, has demonstrated similar performance and utility compared to traditional predictive health-related assessment models [8, 12, 16]. In this study, we investigated the predictive role of the mFI-5 for serious complications and adverse outcomes following radical CRC surgery in elderly patients.

## Materials and methods

### General information

A total of 208 elderly patients, aged ≥ 65 years, who underwent laparoscopic radical surgery for CRC at the Department of Gastroenterology, The Third Affiliated Hospital of Guangzhou Medical College between January 2017 and September 2022, were selected as the study population. The inclusion criteria were as follows: (1) Patients with complete medical history; (2) Patients with no history of other neoplasms; (3) Patients with a postoperative pathological diagnosis of CRC. Exclusion criteria were: (1) Patients undergoing palliative resection or emergency surgery; (2) Patients receiving preoperative neoadjuvant chemotherapy or radiotherapy; (3) Patients with other malignancies. Consent for participation in the study was obtained from the patients themselves or their families, with all participants signing an informed consent form. The radical resections for CRC were performed by our specialist gastrointestinal surgery team. This study was approved by the Ethical Committee of the Third Affiliated Hospital of Guangzhou Medical University and complied with the standards of the Declaration of Helsinki.

### Data collection

The Jiahe Electronic Medical Record System, developed by Beijing Jiahe Meikang Information Technology Co., Ltd, was utilized to extract general preoperative patient data including age, sex, body mass index (BMI) (overweight defined as BMI ≥ 24 kg/m2), hemoglobin concentration (anemia defined as hemoglobin concentration below 120 g/L for men or 110 g/L for women), serum albumin (hypoproteinemia defined as plasma albumin concentration below 30 g/L), history of smoking and alcohol consumption, length of hospital stay, duration of surgery, serious postoperative complications, and mortality within 30 days after surgery. Serious postoperative complications were evaluated based on indicators such as hypoproteinemia, anastomotic leak, incisional infection, pulmonary infection, sepsis, deep vein thrombosis, occurrence of cardiovascular accidents, and readmission to the hospital within 30 days after the operation. Surgical complications were categorized according to the Clavien–Dindo classification (21)—with grades I + II representing minor surgical complications and grades III + IV representing major surgical complications. Perioperative and subsequent complication data was reported for a period of 30 days postoperatively. In this study, the mFI-5 score, as described by Subramaniam et al. [15], was used by accessing the ACS-NSQIP database. The mFI-5 score was treated as a binary categorical variable [15, 18, 25], with an mFI-5 score ≥ 2 indicating frailty and an mFI-5 score < 2 representing the non-frailty group.

### Group setting

A comparison was made between the frail and non-frail groups in terms of age, gender, BMI, serum albumin, hemoglobin, history of smoking and alcohol consumption, tumor location, maximum tumor meridian, as well as surgical data (operative time, intraoperative fluid replacement, intraoperative blood loss) and incidence of postoperative complications (hypoproteinemia, sepsis, pulmonary infections, cardiovascular accidents, lower limb venous thrombosis). Additionally, the mortality rates within 30 days after surgery were compared between groups.

### Statistical analysis

All statistical analyses were performed using SPSS 25.0 software. Continuous variables with normal distributions were presented as mean ± standard deviation (SD), and t-tests were employed for comparison between groups. Continuous variables with skewed distributions were presented as [n (%)], including the number and percentage of cases, and the rank sum test was utilized for comparisons between groups. Categorical variables were expressed as frequencies and percentages within the respective groups, and the *X*^*2*^ test was used for comparison between groups. Multivariate analyses were conducted with frailty as the independent variable and postoperative outcome indicators as the dependent variable. Multivariate logistic and linear regression analyses were employed to adjust for relevant confounders. The predictive value of a mFI-5 score < 2 for postoperative complications was assessed using receiver operating characteristic curve (ROC). A significance level of *P* ≤ 0.05 was considered statistically significant.

## Results

### Serious postoperative complications of patients

The study comprised a total of 208 patients, with 78 individuals experiencing severe postoperative complications, accounting for 38% (78 out of 208). The Clavien Dindo grading system was utilized to categorize the complications. Specifically, complications were classified as I, II, III, IV, and V, representing 22%, 18%, 3%, 7%, and 3% respectively (Table 1). Hypoalbuminemia exhibited the highest incidence rate of serious postoperative complications at 22%. Other complications included lung infection (14%), deep venous thrombosis of the lower limbs (2%), postoperative wound infection (2%), anastomotic fistula (3%), cardiovascular and cerebrovascular accidents (4%), sepsis (3%), and death occurring within 30 days after surgery (3%).

**Table 1:** Clavien-Dindo classification

Classification	Complications	Number	n (%)
I	Hypoproteinemia	46	22
II	Lung infection	30	18
	Lower limb venous thrombosis	5	
	Postoperative wound infection	5	
III	Anastomotic leakage	6	3
IV	Cardiovascular and cerebrovascular accident	9	7
	Sepsis	7	
V	Death within 30 days after surgery	7	3

### Demographic and clinical characteristics of patients

Detailed demographic and clinical information for the 208 patients is presented in Table 2. No significant differences were observed between the two groups concerning preoperative parameters, including gender, BMI, overweight body mass, albumin levels, history of smoking and alcohol consumption, as well as weight loss. Moreover, there were no significant differences observed in surgical parameters such as tumor location, maximum tumor diameter, intraoperative fluid administration, intraoperative bleeding, and operative time (*P* > 0.05).

**Table 2:** Demographic data of patients in two groups

	Total (*n* = 208)	Frailty group (*n* = 73)	Non-frail group (*n* = 135)	*P*-value
Age (years)	72 (67.00–79.75)	75.00 (68.00–83.50)	71.00 (67.00–78.00)	< 0.05
Gender (n [%])				
Male	124 (60)	47 (64)	77 (57)	
Female	84 (40)	26 (36)	58 (43)	0.303
Body mass index (kg/m^2^)	21.9 (19.60–24.10)	21.90 (19.85–24.20)	22.00 (19.40–24.00)	0.471
Combined overweight constitution (BMI ≥ 24 kg/m ^2^) (n [%])	54 (26)	20 (27)	34 (25)	0.728
Weight decreased (n [%])	58 (28)	23 (32)	35 (26)	0.392
Preoperative albumin (g/l)	36.51 ± 4.53	36.11 ± 4.97	36.73 ± 4.28	0.350
Preoperative hemoglobin (g/l)	116.00 (89.50–129.75)	97.00 (82.00–120.00)	120.00 (102.00–131.00)	< 0.05
Smoking history (n [%])	53 (25)	23 (32)	30 (22)	0.142
Alcohol history (n [%])	19 (9)	7 (10)	12 (9)	0.867
Maximum tumor diameter	5.00 (3.50–5.95)	5.00 (3.50–6.00)	4.50 (3.70–5.80)	0.532
Intraoperative bleeding (ml)	20.00 (20.00–50.00)	20.00 (20.00–50.00)	20.00 (20.00–50.00)	0.338
Intraoperative fluid replacement (ml)	2000 (1500–2500)	1550 (1500–2500)	2000 (1500–2450)	0.668
Procedure duration (min)	173.50 (135.00–228.75)	180.00(135.00–240.00)	170.00 (135.00–216.00)	0.453
Total hospital stays (d)	17.00 (14.00–21.00)	19.00 (15.00–24.50)	17.00 (14.00–20.00)	< 0.05
Tumor site (n [%])				
Ileocecal junction (n [%])	4 (2)	2 (3)	2 (1)	
Ascending colon cancer (n [%])	43 (11)	17 (15)	26 (19)	
Transverse colon cancer (n [%])	23 (6)	11 (15)	12 (9)	
Colon cancer descending (n [%])	12 (6)	2 (3)	12 (9)	
Sigmoid colon cancer (n [%])	59 (28)	19 (26)	40 (30)	
Rectosigmoid junction carcinoma (n [%])	6 (3)	2 (3)	4 (3)	
Rectal cancer (n [%])	52 (25)	17 (23)	35 (26)	
Anorectal carcinoma (n [%])	4 (2)	1 (1)	3 (2)	0.765

Age, body mass index, preemptive hemoglobin, maximum tumor diameter, intrapersistent bleeding, intrapersistent fluid replacement, procedure duration and total hospital stay are expressed in median (interquartile interval). Gender,overweight construction, weight reduced, smoking history, alcohol history and tumor site are expressed in %

### Analysis of frailty and serious postoperative complications

#### Univariate analysis of frailty and postoperative complications

As shown in Table 3, patients with mFI-5 ≥ 2 exhibited a higher incidence of serious complications compared to those with mFI-5 < 2 (*P* = 0.001). Specifically, the incidence of complications such as pulmonary infections, sepsis and cardiovascular accidents was significantly higher in the frail group compared to the non-frail group (*P* < 0.05). Additionally, the frail group had a significantly higher incidence of death within 30 days after surgery compared to the non-frail group (*P* = 0.014). Conversely, no statistically significant differences between the two groups were observed in terms of anastomotic leak, postoperative incisional infection, hypoproteinemia, lower limb venous thrombosis, other complications, or return to the hospital within 30 days postoperatively (*P* > 0.05).

**Table 3:** Univariate analysis of frailty and severe postoperative complications

Complication (n [%])	Total (*n* = 208)	Frailty group (*n* = 73)	Non-frail group (*n* = 135)	*P*-value
Postoperative serious complications	78 (38)	38 (53)	40 (30)	0.001
Anastomotic leakage	6 (3)	3 (4)	3 (2)	0.732
Postoperative wound infection	5 (2)	3 (4)	2 (1)	0.48
Hypoproteinemia	46 (22)	20 (27)	26 (19)	0.177
Lung infection	30 (14)	17 (23)	13 (10)	0.007
Lower limb venous thrombosis	5 (2)	3 (4)	2 (1)	0.48
Sepsis	7 (3)	6 (8)	1 (1)	0.014
Cardiovascular and cerebrovascular accident	9 (4)	7 (10)	2 (1)	0.017
Death within 30 days after surgery	7 (3)	6 (8)	1 (1)	0.014
Returned 30 days after surgery	2 (1)	0	2 (1)	0.542

Postoperative serious complications, Anastomotic leakage, Postoperative wound infection, Hypoproteinaemia, Lung infection, Lower limb venous thrombosis, Sepsis, Cardiovascular and cerebrovascular accident, Death within 30 days after surgery and Returned 30 days after surgery are expressed in %

#### Logistic regression analysis of frailty and serious postoperative complications

According to Table 4, the incidence of postoperative complications and mortality within 30 days after surgery was greater in the frail group if not corrected. Even after accounting for gender and preoperative hemoglobin levels, the frail group continued to have a higher risk of postoperative complications and mortality within 30 days compared to the non-frail group. Frailty was an independent risk factor for postoperative pulmonary infections, sepsis, cardiovascular events, and mortality within 30 days after surgery.

**Table 4:** Logistic regression analysis of postoperative complications and 30-day mortality

Model 1	B value	OR	95% CI	*P*-value
Severe postoperative complications	1.059	2.883	1.504—5.528	0.001
Lung infection	1.171	3.226	1.384–7.520	0.007
Sepsis	2.603	13.511	1.513—120.675	0.02
Cerebrovascular accident	1.865	6.456	1.194—34.898	0.03
Death within 30 days	2.601	13.479	1.509—120.394	0.02

Adjusted for age, preoperative hemoglobin, and other variables. OR, odds ratio; CI, confidence interval

### Univariate and multivariate analysis of postoperative complication

The results presented in Table 5 demonstrate significant differences between the group experiencing postoperative complications and the group without such complications. These differences were observed in various factors, including the presence of overweight (BMI ≥ 24 kg/m^2^), preoperative levels of albumin and hemoglobin, frailty (mFI-5 ≥ 2), and the total length of hospital stay (*p* < 0.05). Through binary logistic multivariate analysis, it was determined that frailty (mFI-5 ≥ 2), as well as decreased levels of preoperative serum albumin and hemoglobin, were independent risk factors for postoperative complications. Among these factors, the mFI-5 exhibited the greatest predictive ability for severe postoperative complications (OR = 2.254, 95% CI 1.344—4.740, *P* = 0.004).

**Table 5:** Univariate and multivariate analysis of postoperative Complication

Variable	Univariate analysis		Multivariate analysis	
	OR (95%CI)	*P*-value	OR (95%CI)	*P*-value
Combined overweight (BMI ≥ 24 kg/m^2^)	0.434 (0.215—0.875)	0.020	0.466 (0.223—0.976)	0.043
Preoperative albumin (g/l)	1.110 (1.039—1.186)	0.002	1.083 (1.006—1.165)	0.034
Preoperative hemoglobin (g/l)	1.017 (1.004—1.031)	0.011	1.005 (0.989—1.020)	0.559
Frailty (mFI-5 ≥ 2)	2.579 (1.430—4.649)	0.002	2.254 (1.344—4.740)	0.004

Preoperative albumin is expressed as the mean.OR, odds ratio; CI, confidence interval;Frailty (mFI-5 ≥ 2)

### Predictive power of the predictive value of mFI-5

**Fig. 1:**
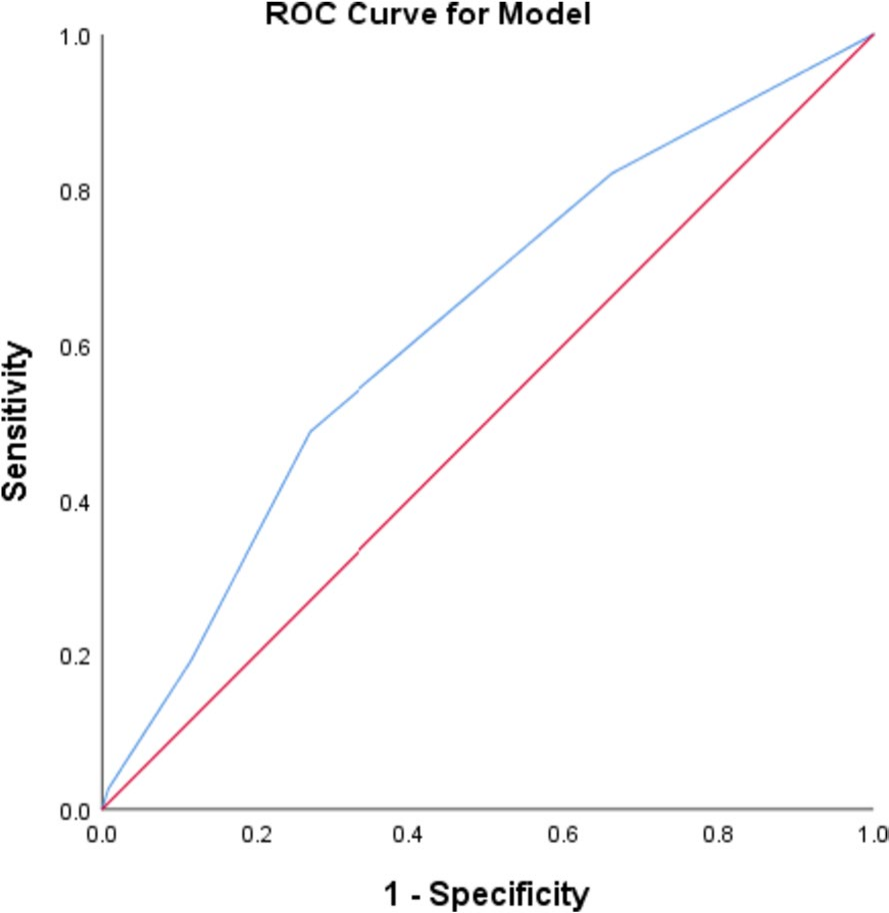
Receiver operating characteristic (ROC) curve for mFI-5 for the prediction of complications following radical resection of colorectal carcinoma in elderly patients

As shown in Fig. 1, the area under the curve of the mFI-5 for the prediction of complications following radical resection of colorectal carcinoma in elderly patients was found to be 0.629 (95% CI: 0.551—0.707) (*P* < 0.05).

## Discussion

In this retrospective analysis of elderly patients undergoing radical CRC surgery, statistically significant differences were found in baseline data between frail and non-frail patients regarding age, preoperative hemoglobin levels, and other variables. Frail patients with an mFI-5 score of ≥ 2 had longer hospital stays compared to the non-frail group. They also experienced significantly higher rates of postoperative pulmonary infections, sepsis, cardiovascular accidents, and other complications, leading to mortality within 30 days after surgery. Multivariate analysis indicated that frailty (mFI-5 ≥ 2), preoperative albumin levels, and prolonged hospitalization were independent risk factors for serious postoperative complications. These findings suggest that mFI-5 plays a crucial role in predicting such complications and short-term postoperative mortality. Similar to previous research on various subspecialties, mFI-5 ≥ 2 has consistently shown beneficial results in studies of radical CRC surgery in elderly patients [26, 27]. Studies in orthopedics, neurosurgery, and other fields have demonstrated that mFI-5 is an independent predictor of prognostic evaluations, including postoperative medical complications and outcomes. It has been shown to be consistent with the findings of the mFI-11 as an independent predictor of postoperative complications in various surgical specialties [12, 15, 16, 26, 27]. Therefore, this present study evaluating CRC surgery in older adults using mFI-5 aligns with previous studies assessing the benefits of CRC surgery in older adults using mFI-11 in large national databases [28].

The results of this study indicate that age of patients correlates with the prognosis of CRC treatment. However, it is important to note that not all elderly patients are frail. Shintaro et al. conducted a study showing that patients in their 90 s can safely undergo radical resection of CRC. Elderly patients who exhibit good preoperative performance can still achieve excellent short-term and long-term prognoses after surgery [29]. The mFI-5 offers rapid identification in clinical practice, allowing for early recognition and improvement of a patient's physiological reserve. This approach can lead to the best possible outcomes for patients with underlying diseases or low functional reserves. For high-risk patients, aggressive preoperative interventions such as exercise, respiratory adjustment training, or nutritional supplementation may enhance their physiological reserve and improve prognosis. A systematic review of 15 randomized trials comparing 457 pretreated patients with 450 controls undergoing major abdominal surgery showed that pretreated patients had lower rates of pulmonary morbidity and postoperative complications [30]. Several studies [31, 32] have demonstrated that preoperative exercise and nutritional therapy can improve frailty, facilitate postoperative recovery from gastrointestinal malignancies, and reduce hospital stays. Optimizing and intervening in the preoperative state benefits both patients and reduces the burden on the medical system. Anastomotic leakage after CRC surgery was found to prolong postoperative hospitalization by 26 days and increase total hospitalization costs by $10,970 compared to cases without leakage [33]. Manuel's study also revealed a correlation between prolonged postoperative hospital stay and postoperative severity [34]. In clinical diagnosis and treatment, particularly in perioperative management, frailty is associated with factors such as malnutrition and inadequate cardiopulmonary reserve, which can be modified. Therefore, incorporating mFI-5 into clinical decision-making helps identify and stratify older patients undergoing radical CRC surgery, enhances understanding of the additional risks associated with increased frailty, improves expectations of the recovery process, and optimizes patient prognosis.

However, this study had several limitations. It was a retrospective clinical study with a small sample size. Due to the lack of large epidemiological surveys and studies on elderly patients undergoing radical CRC in China, the results may not fully represent the overall situation of Chinese patients. Therefore, it is necessary to carry out studies with larger samples in future to further validate these findings. Additionally, data availability was limited to the first 30 days after the operation, which may have underestimated the incidence of complications. Thus, future studies should collect and analyze long-term follow-up data. Nonetheless, this study is the first to use an mFI-5 score of ≥ 2 as a cutoff value for radical CRC surgery in the elderly. This suggests that mFI-5 is promising for rapid preoperative clinical risk stratification.

## Conclusions

MFI-5 was demonstrated to be a reliable predictor of complications and mortality occurring within 30 days after CRC surgery. By combining the preoperative mFI-5 with preoperative albumin levels, it is possible to more accurately identify patients at high risk, thus offering a valuable tool for assessing surgical risk, optimizing planning for elective surgeries, and effectively managing resources in elderly patients undergoing radical CRC surgery.

### Supplementary Information

**Additional file 1.**

## Data Availability

The data and materials contributing to this article may be made available upon request by sending an e-mail to the corresponding author.

## Abbreviations

CRC: Colorectal cancer
mFI: Modified frailty index
CSHA-FI: Canadian Study of Health and Aging-Frailty Index
ACS-NSQIP: American College of Surgeons National Surgical Quality Improvement Program
CHF: Congestive heart failure
COPD: Chronic obstructive pulmonary disease
ROC: Receiver operating characteristic
BMI: Body mass index
mFI-11: 11-Factor Modified Frailty Index
mFI-5: 5-Factor Modified Frailty Index
