# Supplementary material for: Application of MFI-5 in severe complications and unfavorable outcomes after radical resection of colorectal cancer
Source: World J Surg Oncol. 2023 Sep 26;21:307. doi: 10.1186/s12957-023-03186-4 (PMC10521557; doi:10.1186/s12957-023-03186-4)
Supplement: Supplementary file 1 — Additional file 1. [file 12957_2023_3186_MOESM1_ESM.docx]

**Supplementary documents**

**Statistical data of preoperative mFI-5 variables in two groups of patients**

The table presents the preoperative variable data of mFI-5 for the two groups of patients. The two groups of patients showed significant statistical differences in functional status, underlying diseases, etc.

Table. Preoperative variables for mFI-5 (n, %)

|  | Frailty group（n=73) | Non-frail group(n=13) | X2 | P-value |
| --- | --- | --- | --- | --- |
| hypertension | 67（91.8%） | 58（43.0%） | 47.086 | P<0.0001 |
| diabetes | 36（49.3%） | 7（5.2%  ） | 56.264 | P<0.0001 |
| chronic obstructive pulmonary disease (COPD) or pneumonia | 27（37.0%） | 10（7.4%） | 28.346 | P<0.0001 |
| dependent function | 11（15.1%） | 2（1.5%  ） | 14.928 | P<0.0001 |
| congestive heart failure | 35（47.9%） | 2（1.5%  ） | 69.944 | P<0.0001 |

Hypertension, diabetes, chronic obstructive pulmonary disease (COPD) or pneumonia, dependent function and congestive heart failure are expressed in%.
